# Lymphocytes have a role in protection, but not in pathogenesis, during La Crosse Virus infection in mice

**DOI:** 10.1186/s12974-017-0836-3

**Published:** 2017-03-24

**Authors:** Clayton W. Winkler, Lara M. Myers, Tyson A. Woods, Aaron B. Carmody, Katherine G. Taylor, Karin E. Peterson

**Affiliations:** Laboratory of Persistent Viral Diseases, Rocky Mountain Laboratories, National Institute of Allergy and Infectious Diseases (NIAID), National Institutes of Health (NIH), 903 S. 4th St., Hamilton, MT 59840 USA

**Keywords:** Lymphocytes, La Crosse Virus, Brain, Central nervous system, Encephalitis

## Abstract

**Background:**

La Crosse Virus (LACV) is a primary cause of pediatric viral encephalitis in the USA and can result in severe clinical outcomes. Almost all cases of LACV encephalitis occur in children 16 years or younger, indicating an age-related susceptibility. This susceptibility is recapitulated in a mouse model where weanling (3 weeks old or younger) mice are susceptible to LACV-induced disease, and adults (greater than 6 weeks) are resistant. Disease in mice and humans is associated with infiltrating leukocytes to the CNS. However, what cell types are infiltrating into the brain during virus infection and how these cells influence pathogenesis remain unknown.

**Methods:**

In the current study, we analyzed lymphocytes recruited to the CNS during LACV-infection in clinical mice, using flow cytometry. We analyzed the contribution of these lymphocytes to LACV pathogenesis in weanling mice using knockout mice or antibody depletion. Additionally, we studied at the potential role of these lymphocytes in preventing LACV neurological disease in resistant adult mice.

**Results:**

In susceptible weanling mice, disease was associated with infiltrating lymphocytes in the CNS, including NK cells, CD4 T cells, and CD8 T cells. Surprisingly, depletion of these cells did not impact neurological disease, suggesting these cells do not contribute to virus-mediated damage. In contrast, in disease-resistant adult animals, depletion of both CD4 T cells and CD8 T cells or depletion of B cells increased neurological disease, with higher levels of virus in the brain.

**Conclusions:**

Our current results indicate that lymphocytes do not influence neurological disease in young mice, but they have a critical role protecting adult animals from LACV pathogenesis. Although LACV is an acute virus infection, these studies indicate that the innate immune response in adults is not sufficient for protection and that components of the adaptive immune response are necessary to prevent virus from invading the CNS.

## Background

La Crosse Virus (LACV) is a tri-segmented, single-stranded, negative-sense RNA virus of the genus *Orthobunyavirus*, in the *Bunyaviridae* family. The virus is primarily transmitted by the Eastern Tree Hole mosquito (*Ochlerotatus triseriatus*) and was first shown to cause human neurological disease when isolated from the brain of a 4-year girl who died from viral encephalitis in La Crosse, Wisconsin in 1960 [1]. Cases of LACV encephalitis have been reported in 29 states, and the virus has been found in other invasive mosquito species, suggesting this is an emerging disease [2, 3]. LACV is a primary cause of pediatric viral encephalitis in the USA, with almost all cases of LACV-induced neurological disease observed in children under the age of 16. Some of these cases can be severe, with symptoms such as paralysis or coma and in a few cases per year, even death. In contrast, in adults and older teens LACV infection is generally either asymptomatic or causes a mild febrile disease. It does not cause encephalitis, suggesting that in adults virus is effectively cleared following infection.

Currently, there is no vaccine available to inhibit LACV infection [4, 5] or therapy to treat virus-induced encephalitis [2]. Thus, a better understanding of the mediators of viral pathogenesis in the central nervous system (CNS) as well as the mechanisms by which adults are protected from the development of encephalitis is necessary to mitigate disease impact. In patient brain biopsies and animal models of LACV-induced encephalitis, a common feature of disease pathology is infiltration of leukocytes in the brain [6–8]. However, the make-up of this infiltrating population and their potential role in LACV pathogenesis remains unknown. We have previously established that circulating leukocytes do not contribute to virus neuroinvasion [9] as has been suggested for other encephalitic viruses [10, 11]. However, these cells may have other roles in influencing viral pathogenesis. Infiltrating lymphocytes including natural killer (NK) cells, CD8^+^ T cells, CD4^+^ T cells, and B cells are often necessary for viral clearance from the brain, mediate recovery from multiple encephalitic virus infections [12–14] and may protect against LACV-induced neuronal damage. However, these cells can also contribute to neuronal damage, as observed with West Nile encephalitis [15–17]. Determining which cell populations gain access to the CNS during LACV infection, as well as the influence of these cells on disease, is essential for developing therapeutics to inhibit this disease.

The murine model of LACV infection is ideal for studying neuropathogenesis and disease resistance, as it recapitulates the age-dependent susceptibility observed in humans [18, 19]. Following peripheral infection, adult mice are resistant to neurological disease and have low CNS titers [19], while weanlings are susceptible and have high CNS titers [18, 20]. This age-related resistance in adults appears to be due to the inability of LACV to replicate in the periphery and/or invade the CNS, as administration of LACV to the CNS by intracerebral [18] or intranasal inoculation [9] results in neurological disease in adult mice. Suppression of type I interferon (IFN) signaling in the periphery or depletion of myeloid dendritic cells (DCs) in adult animals can result in neurological disease [21, 22], suggesting that an efficient anti-viral type I IFN response is necessary for preventing disease in adult animals. However, it remains unknown whether the innate immune response is sufficient to control LACV infection in adult animals, or if adaptive immune responses, including the production of neutralizing antibodies, are also necessary in preventing the development of neurological disease.

In the current study, we examined the role of different lymphocyte populations in mediating neurological disease following LACV infection in weanling mice. Additionally, we determined if these lymphocytes influenced viral clearance and protection in adult mice. Lymphocyte-deficient transgenic mice and depletion studies demonstrated that lymphocytes did not significantly influence LACV-induced neurological disease in young animals. However, both B and T cells were necessary for efficient virus clearance and the prevention of neurological disease in adult animals. These findings demonstrate that while lymphocytes are not mediators of disease in young susceptible animals, they do provide protection in resistant adult animals. Furthermore, this study indicates that both innate and adaptive immune responses are essential for efficient virus clearance and the prevention of neurological disease following LACV infection.

## Methods

### Infection of mice with LACV and neurological disease criteria

All animal studies were conducted using the animal protocol RML2014-011, which was approved by the NIH/NIAID/RML Institutional Animal Care and Use Committee. Founder wildtype (C57BL/6), *Rag1* ^-/-^, and μ*MT* ^-/-^ mice on the C57BL/6 background were purchased from Jackson Laboratories and maintained in a breeding colony at RML. LACV 1978 stock, a human isolate, was a kind gift from Richard Bennett (NIAID, NIH) and has been previously described [8]. Mice at 3 (weanling) or 6–8 (adult) weeks of age were inoculated with 10^3^ plaque forming units (PFU) of LACV in phosphate-buffered saline (PBS) intraperitoneally (i.p.) in a volume of 200 μl/mouse. Mock infections consisted of an equal volume of Vero cell culture supernatant diluted into PBS. Mice were observed daily for signs of neurological disease that included hunched posture, seizures, reluctance, or inability to move normally or paralysis. Animals with clear clinical signs of neurological disease were scored as clinical and euthanized immediately.

### Treatment of mice with cell-depleting antibodies

For the depletion of T cells, anti-CD8 clone 169.4 and anti-CD4 clone 191.1 hybridomas were grown in RPMI media containing 10% FBS to a concentration of 2 mg/ml as measured by 260/280 absorbance. Supernatants (gift of Dr. Kim Hasenkrug, NIAID, NIH) were harvested and spun at 500 × *g* for 10 min to remove any cellular debris and then stored at −20 °C until use. Weanling mice were injected i.p. with 0.5 ml of the supernatant a total of three times (1, 3, and 5 days post infection (dpi)). Dual CD8 T cell- and CD4 T cell-depleted mice received two injections (a total of 1 ml of supernatant) at each indicated time point. Adult LACV-infected mice followed the same injection schedule with two additional injection days at 12 and 19 dpi. Control mice were injected on the same schedules with 10% FBS in RPMI. T cell depletion was confirmed by flow cytometry using CD3, CD4, CD8a, and CD8b.2 antibodies.

LACV-infected weanling mice were depleted of natural killer (NK)-cells by the i.p. administration of 50 μl of rabbit anti-Asialo-GM1 (Wako) at 1, 3, and 5 dpi. Adult LACV-infected mice received the same injections with an additional injection at 9 dpi. NK cell depletion was confirmed by flow cytometry using NK1.1 and CD49b (clone DX5) antibodies.

### Evans Blue dye treatment

LACV-infected mice were given Evans Blue dye (200 μl of 20 mg/ml intravenously) in PBS at 6 dpi, just prior to the onset of clinical disease. Thirty minutes following dye infusion, mice were perfused transcardially with 5 ml of heparinized saline (100 U/ml) and the brains removed and processed for immunohistochemistry as indicated below. Dye leakage was visualized using epifluorescence microscopy in the TRITC channel.

### Tissues processing for flow cytometry

For phenotypic profiling, verification of T cell depletion studies and lymphocyte activation/proliferation analysis, whole brains from mock and LACV-infected weanling mice were isolated at specific time points and a single-cell suspension made by homogenization and passage through a 70 μm filter. Individual mice were compared to allow determination of variation between animals. Cells were pelleted and resuspended in 70% Percoll/PBS and underlayed on a 0–30% step Percoll gradient which was centrifuged at 500*g* for 20 min at 4 °C. CNS immune cells were recovered at the 30–70% interface, rinsed in PBS, and placed on ice to await fixing or staining. For verification of antibody-mediated cell depletions and lymphocyte-activation/proliferation analysis, the spleens from weanling and adult mice were homogenized through a 70 μm filter to generate a single-cell suspension and red blood cells were removed using 2% dextran T500–PBS and/or lysis buffer (0.15 M NH_4_Cl, 10 mM KHCO_3_, 0.1 M EDTA).

### Phenotyping CNS-infiltrating immune cells and splenocytes by flow cytometry

Cells were isolated as described above and then processed for flow cytometry as previously published [22]. Briefly, cells were fixed in 2% paraformaldehyde and then permeabilized with 0.1% saponin–2% bovine serum albumin (BSA) in PBS. Fc receptors were blocked using CD16/CD32 Fcγ III/II (BD Biosciences, clone 2.4G2). Cells were stained using the following panel of antibodies (all antibodies used for flow cytometry were purchased from BD Pharmigen, BioLegend, Miltenyi, eBiosciences, or Molecular Probes) to establish a lymphocyte phenotype: CD45-PE (30-F11), CD4-APC/Cy7 (GK1.5), CD8a-PB (53-6.7), CD8b.2-FITC (53-5.8), CD3-PerCP/Cy5.5 (UCHT1), CD19-PE-CF594 (1D3), NK1.1-AF700 (PK136), and CD49b (DX5)-PE (DX5). The following antibodies were used in various combinations with the antibodies from the lymphocyte panel to exclude non-lymphocytic cells: CD11c-PE/Cy7 (HL3), pDCA1-APC (JF05-1C2.4.1), CD11b-APC (M1/70), Ly6G-PB (1A8), Ly6C-AF700 (HK1.4), and F480-BV510 (BM8). All flow cytometry data was obtained using an LSRII (BD Biosciences) and analyzed using either FlowJo software (version 10.2; TreeStar, Inc) or FCS Express software (version 3, De Novo). Live cells were retained and doublets excluded using SSC-A and FSC-A gating and then live cells were gated by time to exclude any artifact caused by erratic sample flow.

### Analysis of CNS-infiltrating and splenic lymphocyte activation and proliferation by flow cytometry

To determine lymphocyte activation and proliferation state, CNS-infiltrating cells and splenocytes were isolated as described above and surface labeled for ~30 min at 4 °C. Antibodies used for surface staining were as follows: Pacific Blue-anti-CD8 (53-6.7) and CD4 (RM4-5), APC/Cy7-anti-CD3e (17A2), PerCP-Cy5.5-anti-CD43 (1B11), AF488-anti-CD25 (PC61), FITC-anti-CD107a (1D4B), PE-anti-CD11a (2D7), BV605-anti-KLRG1 (2F1), PE-CF594-anti-PD1 (J43), and PE-Cy7-anti-CD62L (MEL-14). Cells were then fixed overnight using reagents and following recommendations from the eBioscience Foxp3 kit, permeabilized, and then stained intracellularly with APC-anti-Foxp3 (FJK-16s) or APC-anti-Granzyme B (GRB05) and Alexa700-anti-Ki-67 (B56). Cells were then washed and fixed for 30 min at 4 °C with 2% paraformaldehyde. Flow cytometry data was collected as described above. T cells were positively gated by either CD4^+^ or CD8^+^ and CD3^+^ co-expression, and in the case of CD4+ T helper cells, Foxp3 exclusion. T cells were further analyzed for the expression of the phenotyping markers listed above. Splenocytes from the naïve weanlings and adults (and non-specific Ig or FMO controls where appropriate) were used to determine gating placement.

### Immunohistochemistry

**Fig. 1:**
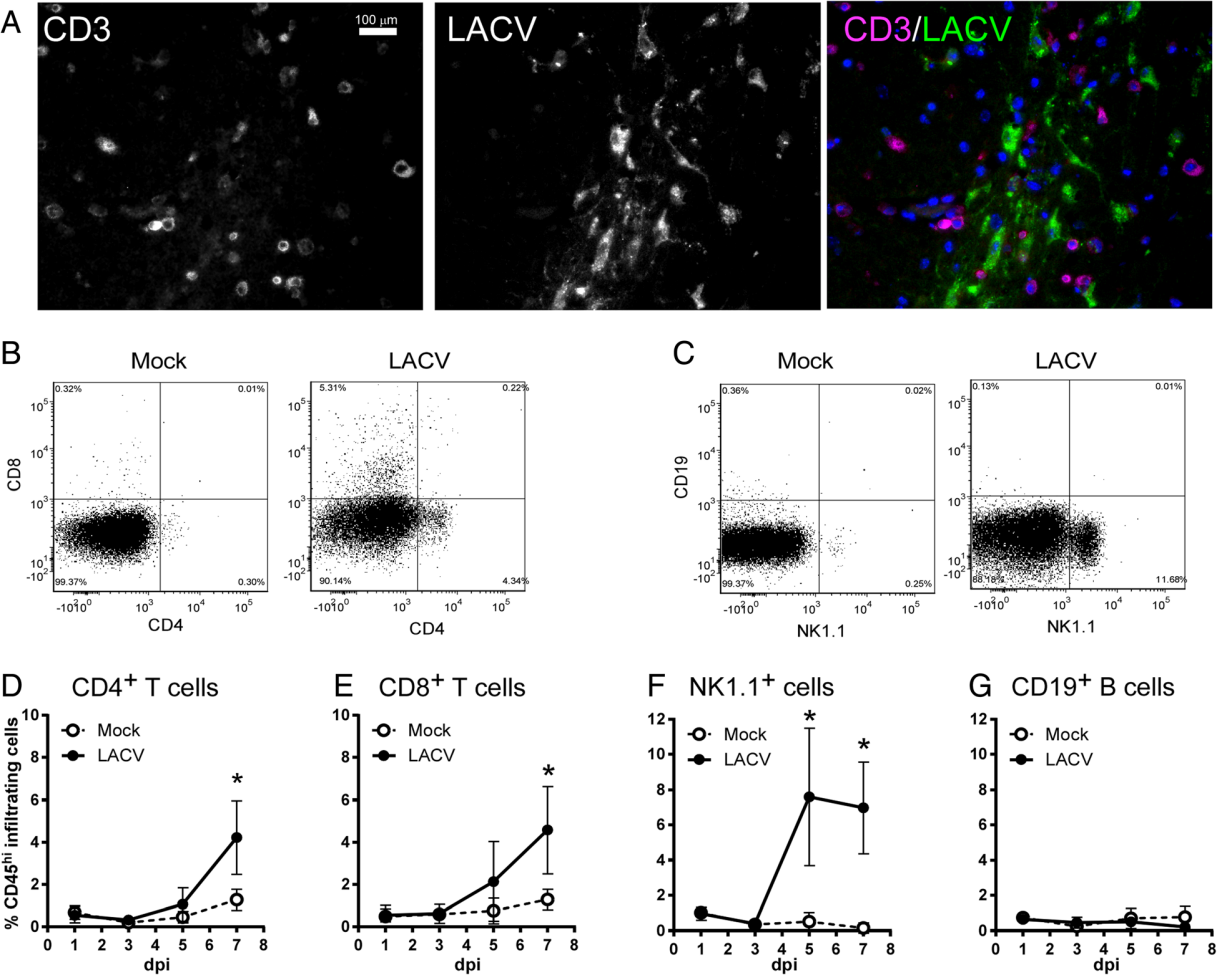
Lymphocyte infiltration into the CNS following LACV infection of weanling mice. **a** Brain tissue sections from an LACV-infected mouse at a clinical time point (7 dpi) was stained for anti-CD3 (*first panel*, *third panel*: *magenta*) and LACV (*second panel*, *third panel*: *green*). **b**–**g** Analysis of infiltrating cells by flow cytometry. Brain tissue was removed from mock or LACV-infected mice at 1, 3, 5, or 7 dpi and immune cells were isolated, antibody labeled, and analyzed by flow cytometry as indicated in the “Methods”. Infiltrating cells were gated by CD45 high expression (data not shown) and then for **b** CD4 and CD8 expression as well as **c** CD19 and NK1.1 expression. **b**, **c** are representative data from mice at 7 dpi. **d**–**g** Time course analysis of percent (%) infiltrating cell populations including **d** CD4^+^ cells, **e** CD8^+^ cells, **f** NK1.1^+^ cells, and **g** CD19^+^ B cells relative to the total number of live, CD45^hi^ cells. Data are the mean ± SD for three to nine samples per time point for LACV-infected mice and one to three mice per time point for mock-infected controls. *P value <0.05 as determined by two-way ANOVA with Sidak’s multiple comparison test

**Fig. 2:**
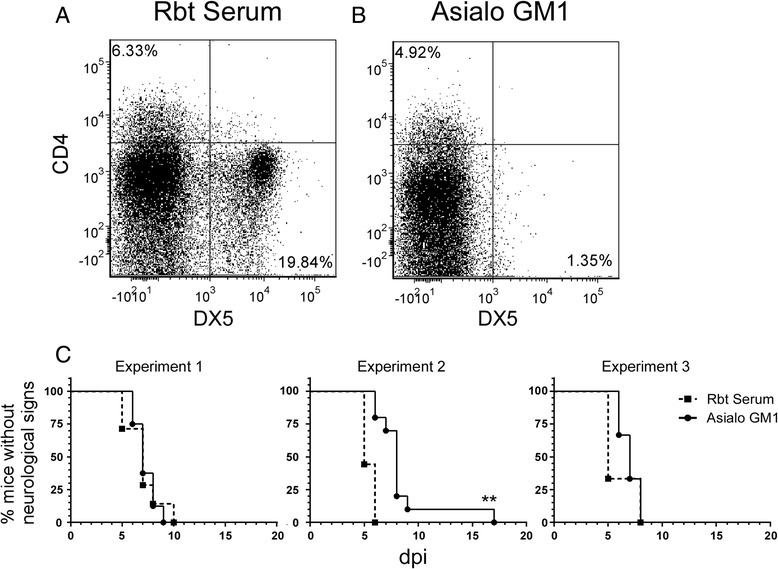
NK cell depletion slightly delays onset of LACV-induced neurological disease in weanling mice. **a**–**c** LACV-infected weanling mice were treated with **a** rabbit serum or **b** anti-Asialo GM1 to deplete NK cells as described in the “Methods.” Flow cytometry analysis at 7 dpi was used to confirm that treatment with **b** anti-Asialo GM1, but not **a** rabbit serum resulted in depletion of NK cells as shown by CD49b (DX5) expression. Experiment 1 included 4–5 mice per group, experiment 2 had 9–10 mice per group, and experiment 3 had 3 mice per group. Statistical analysis was completed using the Mantel-Cox log-rank test

At the clinical time point (6–7 dpi), some mice were perfused transcardially with heparin saline (100 U/ml) followed by 10% neutral buffer formalin. The whole brain was serially sectioned (5 μm), and sections were blocked (5% BSA, 0.05% Triton in PBS) at room temperature (rt) for 1 h. Primary antibodies against CD3 (1:250, Dako), LACV (1:1000, gifted by Dr. Robert Tesh) and active-Caspase 3 (1:250, Promega) were applied to sections and incubated overnight at 4 °C in blocking buffer. Secondary antibodies were used to label these specific primaries (donkey anti-rabbit AF488 and goat anti-mouse AF594) and to visualize IgG leakage (goat anti-mouse AF488) in the CNS. Secondary antibodies were incubated for 1 h at rt. Slides were cover slipped with Prolong Gold mounting media containing DAPI (Molecular Probes) and imaged using (1) an epifluorescent Nikon Eclipse 55i clinical microscope with a Plan Fluor ×40 objective (NA 0.75) to generate single images (Figs. 1a and 4a, b) or (2) an Aperio ScanScope FL (fluorescent) slide scanner (Leica Biosystems) with a UPLSAPO ×20 objective (NA 0.75) to generate composite images (Fig. 4e–h).

### Viremia and neutralizing antibody detection

For detection of viremia, serially diluted plasma was plated directly to Vero cells as described below. For quantification of neutralizing antibody, serially diluted plasma was mixed with 10^2^ PFU of LACV in a final volume of 200 μl in DMEM/2% FBS/1% Pen Strep. The mixture was incubated for 1 h at 37 °C for neutralization. After neutralization, the 200 μl mixture was added to confluent Vero cells in a 24-well plate and incubated again for 1 h at 37 °C. After incubation, 500 μl of 1.5% carboxymethyl cellulose in MEM was overlaid onto the cells and the cells were incubated undisturbed at 37 °C for 5 days. Cells were then fixed by adding 10% formaldehyde to each well until full and allowed to sit for 1 h at room temperature. After fixation, plates were rinsed gently with deionized water and stained with 0.35% crystal violet for 15 min. Plates were rinsed and allowed to air dry inverted. Viral titer was calculated by dividing the number of plaques per given sample by the plasma dilution factor multiplied by the volume of each well. Neutralizing antibody titer was determined by the dilution that inhibited at least 50% of plaque formation when compared to cells infected with the 10^2^ LACV.

### Real-time PCR

Real-time PCR analysis of mRNA expression was completed as previously described [23]. The primers used include Gapdh.2-152F (TGCACCACCAACTGCTTAGC), Gapdh.2-342R (TGGATGCAGGGATGATGTTC), LACVs.2-552F (ATTCTACCCGCTGACCATTG), and LACVs.2-650R (GTGAGAGTGCCATAGCGTTG). Primers were subjected to BLAST analysis (NCBI) to ensure detection of only the specified gene and were tested on positive controls to ensure amplification of a single product. Data for each sample were calculated as the percent difference in threshold cycle (*C*_*T*_) value (Δ*C*_*T*_ = *C*_*T*_ for glyceraldehyde-3-phosphate dehydrogenase [GAPDH] gene − *C*_*T*_ for specified gene). Gene expression was plotted as the percentage of gene expression relative to that of the GAPDH gene.

### Statistical analysis

All statistical analyses were performed using Prism software Version 7.01 (GraphPad) and are described in the figure legends.

## Results

### Lymphocyte infiltration into the CNS is associated with areas of LACV infection in weanling mice

Encephalitis associated with LACV infection in both mice and humans is in part characterized by perivascular infiltration of leukocytes into the CNS [7, 8], although the makeup of the infiltrate is not known. Immunohistochemical analysis of brain tissue sections from LACV-infected weanling mice showed CD3^+^ T cells in areas of LACV-infected neurons at the time of clinical disease (Fig. 1a). To determine lymphocyte subtypes and when they first entered the CNS, we analyzed cellular infiltrate in the CNS by flow cytometry at pre- and clinical time points using a lymphocyte-specific antibody panel described in the “Methods”. Although the majority of infiltrating CD45^hi^ cells in clinical mice were CD11b^+^ positive myeloid cells (data not shown), CD4^+^ T cells, CD8^+^ T cells, and NK1.1^+^ lymphocytes were consistently found in the CNS (Fig. 1b, c). CD8^+^ and CD4^+^ T cells were increased in the CNS relative to mock controls at the onset (~7 dpi) of LACV-induced neurological disease (Fig. 1d, e) while CD19^+^ B cells were not detected in the CNS at any point during LACV infection (Fig. 1g). NK1.1^+^ NK cells were significantly increased in the CNS at the pre-clinical 5 dpi time point and remained elevated through to clinical disease (Fig. 1f). Thus, lymphocyte infiltration of the weanling brain occurs during LACV encephalitis and could play a critical role in neurological disease.

### NK cells are minor contributors to LACV-induced pathogenesis in weanling mice

NK cells were the earliest and most abundant lymphocyte to infiltrate the CNS during LACV-induced neurological disease (Fig. 1f). To directly assess the potential role of these cells in the disease process, NK cells were systemically depleted in LACV-infected weanling mice using Asialo-GM1 rabbit antisera. Flow cytometry analysis demonstrated depletion was effective in inhibiting NK cell recruitment to the brain during clinical disease (Fig. 2a, b). However, depletion of NK cells appeared to have little to no effect on disease development, with no statistical difference observed in two experiments (Fig. 2c, experiments 1 and 3) and a small delay of a few days in one other (Fig. 2c, experiment 2). Thus, NK cells may have a minor role in promoting LACV-induced neurological disease but are not essential for viral pathogenesis.

### Infiltrating CD4^+^ and CD8^+^ T cells are proliferative and express effector cell markers but do not contribute to LACV-induced neurological disease

**Fig. 3:**
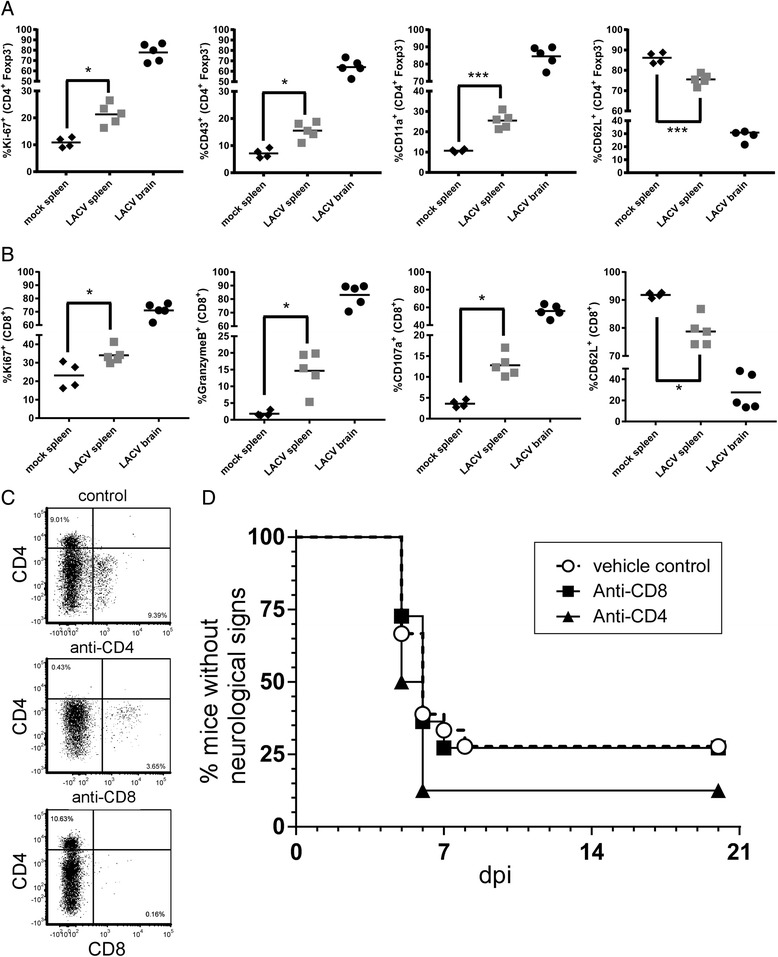
CNS-infiltrating T cells are proliferating and activated during LACV infection but do not significantly alter LACV pathogenesis in weanling mice. Splenocytes and CNS-infiltrating **a** CD4^+^, Foxp3^−^ T helper, and **b** CD8^+^ CD3^+^ cytotoxic T cells were analyzed for expression of proliferation and activation markers by flow cytometry at the clinical time point (6–7 dpi). Data are presented as percent (%) of either CD4^+^ or CD8^+^ T cells positive for the proliferation marker Ki-67, the activation markers CD43, CD11a, GranzymeB, or CD107a or the naïve T cell marker CD62L. **c** LACV-infected weanling mice were treated with RPMI/10% FBS (control), anti-CD4 or anti-CD8-depleting monoclonal antibodies as described in the “Methods”. Depletions were confirmed by flow cytometry analysis of brain tissue at the clinical time point (5–7 dpi). Data are a summary plot of two independent experiments with 4–12 mice per group. **d** 18 vehicle control-, 11 anti-CD8-, and 8 anti-CD4-treated mice were followed for the development of clinical signs of neurological disease. Statistical analysis was completed using the Mantel-Cox log-rank test

CD4^+^ and CD8^+^ T cells have been shown to influence pathogenesis and virus clearance associated with encephalitic viruses [12, 15, 24–26]. Since both CD4^+^ and CD8^+^ T cells were recruited to the CNS during LACV infection, we examined the activation and proliferation markers of these cells and compared them to splenocytes from LACV and mock-infected animals (Fig. 3a, b). There was a higher percentage of Ki67^+^, CD43^+^, and CD11a^+^ CD4^+^ Foxp3^−^ helper T cells in the spleens of LACV-infected mice compared to mock-infected controls (Fig. 3a) consistent with the induction of a strong CD4^+^ T cell response in the periphery. There was also a lower percentage of helper T cells expressing the naïve T cell marker CD62L (Fig. 3a). Analysis of infiltrating cells in the brain indicated that most CD4^+^ T cells had an active phenotype with 50–90% of the cells positive for Ki67, CD43, and CD11a, while less than 40% of the cells were positive for CD62L (Fig. 3a). Similar results were observed with CD8^+^ T cells in regards to proliferation and activation markers, including Ki67 and CD107a (Fig. 3b). Of particular interest, greater than 65% of infiltrating CD8^+^ T cells were positive for Granzyme B (Fig. 3b), suggesting these cells are capable of cytotoxic effector function. Similar to CD4^+^ T cells, the percentage of CD8^+^ T cells expressing the naïve T cell marker CD62L was decreased, correlating with an increase in activated CD8^+^ T cells (Fig. 3b). Brains from mock-infected mice had too few infiltrating T cells to analyze by flow cytometry.

To determine a role for these infiltrating, activated T cells in viral pathogenesis, we depleted each cell type with monoclonal antibodies in weanling mice prior to LACV infection. Flow analysis of infiltrating cells in the brain demonstrated efficient depletion of each cell type with their respective antibodies (Fig. 3c). A slight decrease in CD8^+^ T cells in the CNS was observed in anti-CD4-treated mice, suggesting that CD4 depletion may affect the CD8^+^ T cell recruitment to the CNS (Fig. 3c). However, depletion of either T cell subtype in weanling mice did not significantly alter the occurrence or onset of neurological disease following LACV infection (Fig. 3d), suggesting that T cells are not involved in either the development of neurological disease or in inhibiting its progression.

### IgG antibody is present in the CNS during LACV-induced neurological disease in weanling mice but does not alter neurological disease

**Fig. 4:**
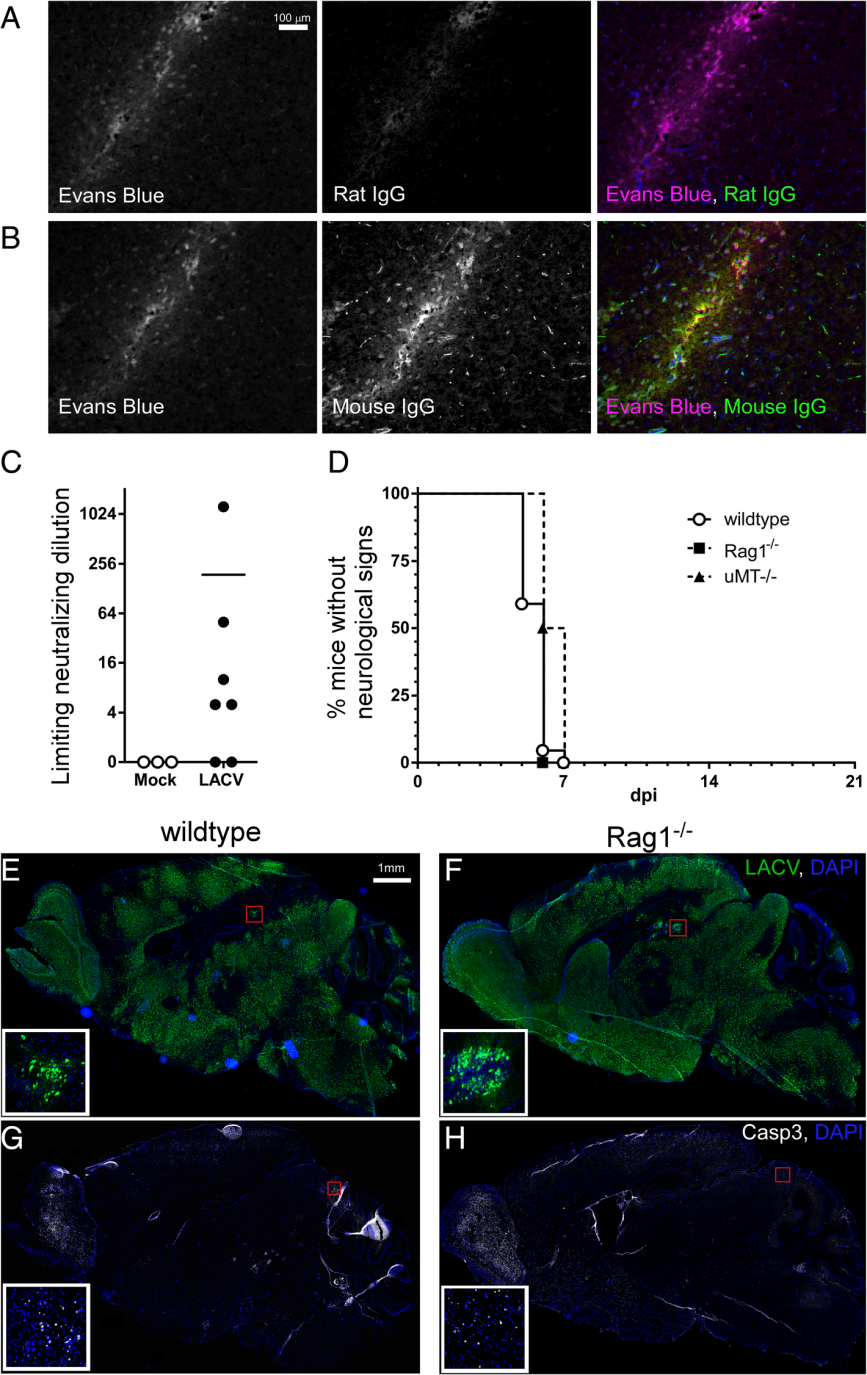
Immunoglobulin can be detected in the brain of LACV-infected weanling mice but does not influence the development of neurological disease or neuropathology. **a**, **b** Detection of antibody in the brain by immunohistochemistry of LACV-infected weanling mice at the clinical time point (6 dpi) that were treated i.p. with Evans Blue dye 1 h prior to tissue removal. Brain tissue sections were analyzed for Evans Blue to detect vascular leakage (*magenta*) and either **a** rat Ig as a negative control or **b** mouse IgG (*green*) was used to detect leakage of antibodies into the brain. **c** Analysis of plasma from LACV-infected weanling mice at 4–5 dpi for NAb. Data are plotted as the limiting dilution for the inhibition of virus replication on a log2 scale. Each symbol represents an individual animal. **d** Weanling wildtype (*n* = 22), μ*MT* ^-/-^ (*n* = 6), and *Rag1* ^-/-^ (*n* = 10) mice develop neurological disease with similar rate and frequency. All mice were 3 weeks of age when infected i.p. with 10^3^ PFU of LACV and followed for clinical disease. Statistical analysis was completed using the Mantel-Cox log-rank test with no significant difference detected. Representative, adjacent sections of brain tissue from **e**, **g** LACV-infected weanling wildtype and **f**, **h** *Rag1* ^-/-^ mice with neurological disease (6–7 dpi) were stained for LACV (*green*) and active-Caspase 3 (*white*). Higher magnification *insets* in **e** and **f** show infected cells (LACV: *green*, DAPI: *blue*) with neuronal morphology within the hippocampus (*red boxes*). *Insets* in **g** and **h** demonstrate cells undergoing apoptosis (active-Caspase 3, *white*) within similar regions of the midbrain (*red boxes*) in both genotypes

The lack of detectable B cells in the CNS during disease could be due in part to isolation methods used or the loss of CD19 when B cells become plasma cells. Additionally, even though these cells were not detected in the brain, they could still be influencing pathogenesis through actions elicited by soluble immunoglobulin (Ig) entering the brain [27, 28]. Blood-brain barrier breakdown occurs in areas of LACV infection [9], which may allow anti-LACV antibodies to enter the CNS. Immunohistochemistry for mouse IgG in the CNS was completed using LACV-infected mice that had received Evans Blue dye to identify areas of a blood-brain barrier leakage (Fig. 4a). IgG was found within the brain parenchyma of these mice but only in areas coinciding with Evans Blue stain (Fig. 4b). As a neutralizing antibody was observed in the plasma from LACV-mice as early as 4–5 dpi (Fig. 4c), the IgG detected in these brains could have the capacity to neutralize virus and affect disease progression.

To directly determine whether antibody responses were important for LACV pathogenesis, B cell deficient weanling μ*MT* ^-/-^ mice [29] were infected with LACV and followed for neurological disease. No difference was observed in either clinical disease development (Fig. 4d) or virus infection in the brain (not shown) in weanling μ*MT* ^-/-^ mice compared to age-matched wildtype controls. Thus, despite the detection of IgG in the CNS of LACV-infected mice, the antibody response did not appear to influence LACV pathogenesis in susceptible weanling mice.

### Combined B cell and T cell deficiency does not influence LACV-induced neurological disease in weanling mice

Despite the fact that B cells (Fig. 4d) and T cells (Fig. 3d) were not individually necessary for the development of LACV-induced neurological disease, it remained possible that the loss of both cellular and humoral adaptive immune responses could influence the development of LACV-induced neurological disease. Therefore, weanling *Rag1* ^-/-^ mice, which are deficient in both mature B and T cells [30], were infected with LACV and monitored for neurological symptoms. No difference was observed in disease onset or occurrence between wildtype and *Rag1* ^-/-^ mice (Fig. 4d) and virus infection was evident in all brain regions in both groups of mice as demonstrated by immunohistochemistry (Fig 4e, f). Also, cellular apoptosis was evident in similar regions of the brain in both wildtype and *Rag1* ^-/-^ mice, suggesting wide-spread neuronal death and neuropathology (Fig. 4g, h). Collectively, these findings demonstrate that the adaptive immune response does not appear to play a significant role in mediating LACV-induced neurological damage in young mice despite the presence of lymphocytes within the CNS.

### Adaptive immune responses are necessary for peripheral control of virus infection in adult mice

**Fig. 5:**
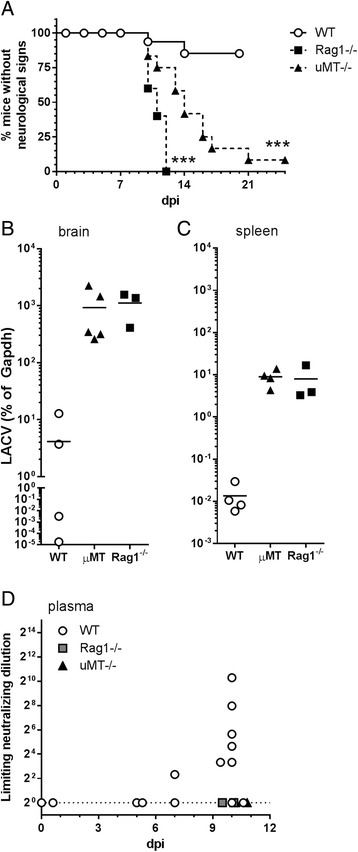
Adult *Rag1* ^-/-^ and μ*MT* ^-/-^ mice have increased susceptibility to LACV infection. **a**–**d** Adult (6–8 weeks old) wildtype, *Rag1* ^-/-,^ and μ*MT* ^-/-^ mice were infected with 10^3^ PFU of LACV and examined for **a** development of neurological disease, **b**, **c** virus RNA levels in the **b** brain and **c** spleen as well as the production of **d** neutralizing antibodies. **a** Data are presented as survival curve analysis of wildtype (*n* = 37), *Rag1* ^-/-^ (*n* = 5), and μ*MT* ^-/-^ (*n* = 12) mice. Statistical analysis was completed using the Mantel-Cox log-rank test. *** *P* <0.01. **b, c** RNA isolated from wildtype, *Rag1* ^-/-,^ and μ*MT* ^-/-^ mice at 10–12 dpi was assayed for LACV RNA by real-time PCR. Data were analyzed as described in “Methods” and are presented as a ratio of expression relative to housekeeping gene for each sample. Each symbol represents an individual mouse. **d** Neutralizing antibodies were detected at 5–10 dpi in wildtype mice. Data are presented as the dilution of plasma resulting in 50% inhibition of virus infection on a log2 scale. No detectable neutralizing antibodies were observed in *Rag1* ^-/-^ or μ*MT* ^-/-^ mice

The lack of a role for lymphocytes in LACV pathogenesis within the CNS in young mice does not rule out a potential role for these cells in controlling peripheral infection and preventing virus entry to the CNS. Adult (6–8 weeks) mice are relatively resistant to LACV-induced neurological disease with less than 25% of wildtype mice developing clinical signs [22]. Previous studies had identified a strong role for early type I IFN responses in controlling virus replication in adult animals, but the impact of the adaptive immune response in preventing the development of neurological disease in these mice had not been examined. Infection of adult *Rag1* ^-/-^ mice resulted in the onset of clinical neurological disease within 10–12 dpi in 100% of infected mice, compared to less than 20% of wildtype mice (Fig. 5a). This increase in disease was associated with higher levels of detectable virus in both the brain and spleen of *Rag1* ^-/-^ mice (Fig. 5b, c). Thus, a functional lymphocyte response was necessary to control peripheral virus infection and to prevent development of LACV encephalitis in disease-resistant adult mice.

### Neutralizing antibody producing B cells are essential for controlling LACV infection

Analysis of wildtype mice indicated the production of anti-LACV neutralizing antibody occurred in most mice by 7–10 dpi (Fig. 5d). As expected, u*MT* ^-/-^ and *Rag1* ^-/-^ mice did not produce neutralizing antibody (Fig. 5d). To determine whether B cells and neutralizing antibodies were essential for protection from LACV-induced neurological disease, we infected μ*MT* ^-/-^ mice with 10^3^ PFU of LACV. Most, although not all, μ*MT* ^-/-^ mice developed neurological disease compared to controls indicating B cells are required for efficient control of LACV infection (Fig. 5a). Susceptibility correlated with detectable viral RNA in the brain and the spleen of u*MT* ^*-*/-^ mice, which were, comparable to viral RNA levels observed in *Rag1* ^-/-^ mice (Fig. 5b). Thus, humoral immune responses are necessary for protection against LACV-induced neurological disease in adult animals.

### Both CD4 and CD8 T cells are necessary for neurological disease resistance

**Fig. 6:**
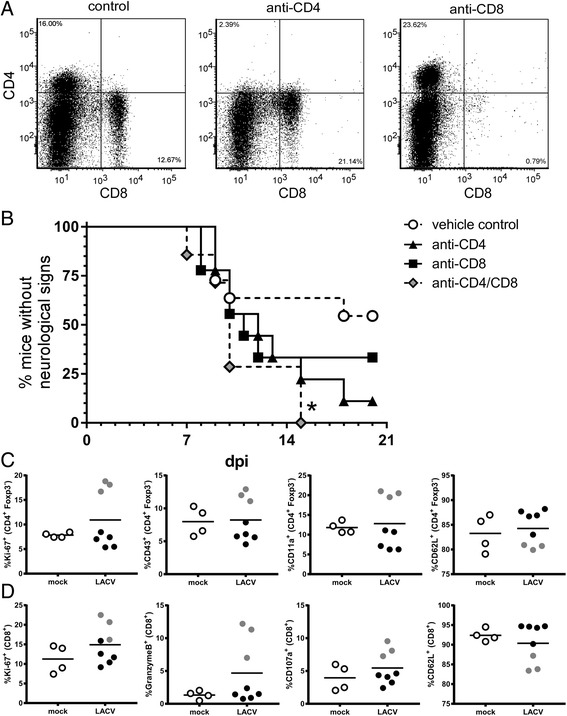
CD4 and CD8 T cells contribute to protection against LACV-induced disease in adult mice. **a**, **b** LACV-infected adult mice were treated with RPMI/10% FBS as a vehicle control, anti-CD4, or anti-CD8 as described in the “Methods”. **a** CD4 and CD8 depletion were confirmed by flow cytometry analysis of splenocytes. **b** Neurological disease development from vehicle-treated controls (*n* = 11), anti-CD4-treated (*n* = 9), anti-CD8-treated (*n* = 9), and anti-CD4/anti-CD8-treated (*n* = 7) mice. Statistical analysis was completed using the Mantel-Cox log-rank test. Although individual anti-CD4 or anti-CD8-treated mice had slightly increased incidence of neurological disease, only depletion of both subsets resulted in a significant (* *P* < 0.05) increase in neurological disease. **c** CD4^+^, Foxp3^−^ T helper, and **d** CD8^+^ CD3^+^ cytotoxic T cells from the spleens of adult mice at 7 dpi were analyzed for expression of proliferation and activation markers by flow cytometry. Data are presented as percent (%) of either CD4^+^ or CD8^+^ T cells positive for the proliferation marker Ki-67, the activation markers CD43, CD11a, GranzymeB, or CD107a or the naïve T cell marker CD62L. Gray circles indicate a group of three mice that showed consistent activation of CD4 and CD8 T cell responses, while black circles indicate mice that did not have increased responses

To examine the specific roles of CD4^+^ T cells and CD8^+^ T cells in LACV clearance and prevention of viral encephalitis, these cell types were depleted from adult wildtype mice prior to infection with LACV (Fig. 6a) using monoclonal antibodies. Depletion of CD4^+^ or CD8^+^ T cells individually slightly increased the number of mice that developed neurological disease (Fig. 6b) compared to vehicle-treated controls. However, when both CD4^+^ and CD8^+^ cells were depleted, all treated mice developed neurological disease suggesting these cells act synergistically to suppress LACV infection (Fig. 6b).

Since CD4^+^ and CD8^+^ T cells suppressed neurological disease in adult mice, we examined these cells for activation and proliferation markers. Mice were analyzed at 7 dpi, the earliest time point of disease in anti-CD4/anti-CD8-treated mice (Fig. 6b). In three of eight mice, an increase in CD4^+^ helper T cells expressing Ki-67, CD43, and CD11a was observed (Fig. 6c, gray circles). These same mice had increased percentages of CD8^+^ T cells expressing Ki67, Granzyme B, and CD107a and a decrease in expression of CD62L (Fig. 6d). Thus, LACV infection in adult mice did not induce a consistently robust CD4^+^ or CD8^+^ T cell response by 7 dpi, with only a subset of animals showing a measurable increase in T cell proliferation and activation. However, these cells are still essential for protection as depletion of both cell types resulted in all animals developing neurological disease (Fig. 6b), suggesting that a more robust response occurs later in infection or that a modest response is sufficient to clear virus.

### NK cells do not contribute to LACV clearance

**Fig. 7:**
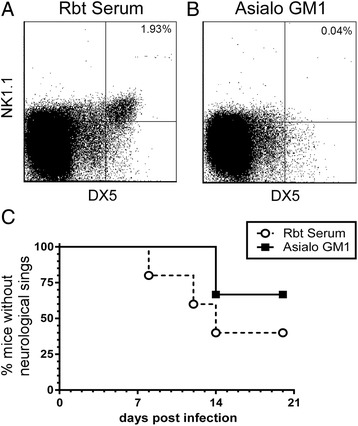
NK cell depletion does not affect susceptibility in adult mice. **a**–**c** LACV-infected adult mice were treated with **a** rabbit serum or **b** anti-Asialo GM1 to deplete NK cells as described in the “Methods”. Flow cytometry analysis was used to confirm that treatment with **b** anti-Asialo GM1, but not **a** rabbit serum resulted in depletion of NK cells. **c** NK cell depletion did not significantly affect development of neurological disease. Data are plotted as a survival curve analysis of five rabbit serum-treated and six anti-Asialo GM1-treated mice. Statistical analysis was completed using the Mantel-Cox log-rank test. No statistical difference was observed

When activated, NK cells can contribute to viral clearance through a cytotoxic mechanism [31]. To determine if NK cells contribute to LACV clearance by the peripheral immune response, adult wildtype mice were infected with LACV and then treated with NK cell-depleting Asialo-GM1 or a control antibody (Fig. 7). Depletion of NK cells was confirmed using DX5 and NK1.1 markers (Fig. 7a). NK cell-depleted mice were as resistant to the development of LACV-induced neurological disease as control-treated mice (Fig. 7b) indicating that NK cells are not essential for protection against LACV infection. Thus, NK cells did not appear to have a major role in LACV pathogenesis, either during disease development in weanling mice or in protection in adult mice.

## Discussion

Anti-viral type I interferon (IFN) signaling is important for acute virus infections. Our previous studies have found that weanling mice that produce low levels of type I IFN do not efficiently control LACV replication early during peripheral infection and go on to develop neurological disease. By contrast, disease-resistant adult mice produce a robust type I IFN response that suppresses measurable viral RNA out to 4 dpi [22]. However, by 5 dpi, viral RNA is significantly increased in the spleens of adult mice, suggesting that a low level of viral replication is taking place [22] and that other mechanisms of protection are needed. In the current study, we show that lymphocytes are necessary for efficient control of peripheral viral infection and the prevention of neurological disease. In contrast, lymphocytes do not appear to have a role in weanling mice, either in preventing disease development or contributing to pathogenesis.

Our current findings demonstrate that LACV infection is not completely controlled by the type I IFN response and that the adaptive immune response is necessary, through both T cell and B cell responses, to inhibit virus replication and prevent neurological disease. Interestingly, the onset of disease in *Rag1* ^-/-^, μ*MT* ^-/-^, or anti-CD4/anti-CD8-treated adult mice generally occurred after 10 dpi and ranged from 7–20 dpi (Figs. 5 and 6). In contrast, disease induced in weanling mice (Figs. 3 and 4) or in adult mice with deficient innate immune responses [22] had a general onset between 5 and 10 days. This delay in disease onset in *Rag1* ^-/-^, μ*MT* ^-/-^, or anti-CD4/anti-CD8-treated adult mice may be due to the ability of the type I IFN response to suppress early virus replication for a short time, resulting in a longer time period for the virus to reach the CNS and induce damage. This low-level prolonged virus replication appears to be controlled by the development of the adaptive immune response, requiring both neutralizing antibodies and T cell responses. How long LACV infection can persist in resistant adult mice without the development of CNS disease and what cell type is infected in the periphery are questions that remain to be addressed more fully to understand the role of the adaptive immune response in LACV pathogenesis.

In contrast to the clear role of lymphocytes in protection against LACV infection in adult animals, there was no substantial effect of depletion or deficiency in lymphocyte function on the development of neurological disease in weanling mice. This finding was surprising, as infiltrating lymphocytes influence pathogenesis in several other cases of viral encephalitis by either clearing infection [15–17] or exacerbating disease [13, 14]. Furthermore, analysis of these cells in LACV-infected weanling mice shows a strong activation phenotype, both in the brain and in the spleen. This contrasts with the results observed in adult mice infected with LACV, where CD4^+^ and CD8^+^ T cells are important for protection, but only a subset of mice had detectable T cell responses at 7 dpi. Thus, the inability of CD4^+^ and CD8^+^ T cells to affect LACV-induced neurological disease in weanling mice is not due to an inability of these cells to respond to virus infection. Indeed, the strong T cell response in weanling mice may be due to the higher level of virus replication, and resulting viral antigen, in the younger mice compared to the adults.

An unanticipated result of treating adult mice with vehicle control reagents containing normal fetal calf or rabbit serum was a trend toward increased incidence of LACV neurological disease (Figs. 6b and 7c). This suggests that heterologous serum elements are in some way increasing overall viral infection. Possible mechanisms include enhanced viral replication or infection, prolonged viral half-life, suppression of host immune response, or enhanced viral entry into the brain. Little published evidence exists to support the idea that heterologous serum facilitates viral replication or infection; however, this possibility cannot be discounted. By contrast, it is unlikely that serum elements are enhancing viral neuroinvasion due to the fact that weanling mice treated with the same serum-rich reagents did not have faster disease onset than untreated LACV-infected weanling mice (Figs. 2 and 3 and [22]). Instead, it is more likely that injection of mice with a low dose of heterologous serum is inducing a modest “immunological paralysis” through a mechanism involving suppression of lymphocyte reactivity [32]. This effect, however, is minor as most control-treated adult mice remain resistant to LACV neurological disease.

## Conclusions

In conclusion, the adaptive immune response, including the cellular response of CD4^+^ and CD8^+^ T cells as well as the humoral B cell response, does not appear to have a critical role in LACV-mediated damage in the CNS in disease-susceptible weanling animals. However, both humoral and cellular adaptive immune responses are critical for disease protection in adult animals. It is possible the more mature and presumptively more efficient adaptive response [33] in resistant adult animals compared to susceptible weanling mice is responsible for this disparity in disease resistance. Furthermore, the adaptive immune response may be more suited to controlling LACV infection in a minor cell population in the periphery, rather than a massive infection of neurons in the CNS. Indeed, when adult mice are infected either through the intranasal route or by direct intracranial injection, they develop neurological disease with similar speed and frequency as weanling mice despite having a fully developed adaptive immune response [9, 22]. This insufficiency of response may be related in part to lymphocyte accessibility to the brain compared to the peripheral tissues as well as to the extremely responsiveness of the brain to injury [34]. Thus, enhancing the peripheral adaptive immune response against LACV in susceptible individuals may be protective against the development of neurological disease by clearing infection prior to the virus entering the CNS where infection cannot be efficiently controlled.

## References

[1] Thompson WH, Kalfayan B, Anslow RO (1965). Isolation of California encephalitis group virus from a fatal human illness. Am J Epidemiol.

[2] McJunkin JE, Nahata MC, De Los Reyes EC, Hunt WG, Caceres M, Khan RR, Chebib MG, Taravath S, Minnich LL, Carr R (2011). Safety and pharmacokinetics of ribavirin for the treatment of la crosse encephalitis. Pediatr Infect Dis J.

[3] Westby KM, Fritzen C, Paulsen D, Poindexter S, Moncayo AC (2015). La Crosse encephalitis virus infection in field-collected Aedes albopictus, Aedes japonicus, and Aedes triseriatus in Tennessee. J Am Mosq Control Assoc.

[4] Schuh T, Schultz J, Moelling K, Pavlovic J (1999). DNA-based vaccine against La Crosse virus: protective immune response mediated by neutralizing antibodies and CD4+ T cells. Hum Gene Ther.

[5] Bennett RS, Gresko AK, Nelson JT, Murphy BR, Whitehead SS (2012). A recombinant chimeric La Crosse virus expressing the surface glycoproteins of Jamestown Canyon virus is immunogenic and protective against challenge with either parental virus in mice or monkeys. J Virol.

[6] McJunkin JE, de los Reyes EC, Irazuzta JE, Caceres MJ, Khan RR, Minnich LL, Fu KD, Lovett GD, Tsai T, Thompson A (2001). La Crosse encephalitis in children. N Engl J Med.

[7] McJunkin JE, Khan R, de los Reyes EC, Parsons DL, Minnich LL, Ashley RG, Tsai TF (1997). Treatment of severe La Crosse encephalitis with intravenous ribavirin following diagnosis by brain biopsy. Pediatrics.

[8] Bennett RS, Cress CM, Ward JM, Firestone CY, Murphy BR, Whitehead SS (2008). La Crosse virus infectivity, pathogenesis, and immunogenicity in mice and monkeys. Virol J.

[9] Winkler CW, Race B, Phillips K, Peterson KE (2015). Capillaries in the olfactory bulb but not the cortex are highly susceptible to virus-induced vascular leak and promote viral neuroinvasion. Acta Neuropathol.

[10] Miller F, Afonso PV, Gessain A, Ceccaldi PE (2012). Blood-brain barrier and retroviral infections. Virulence.

[11] Suen WW, Prow NA, Hall RA, Bielefeldt-Ohmann H (2014). Mechanism of West Nile virus neuroinvasion: a critical appraisal. Viruses.

[12] Griffin DE (2003). Immune responses to RNA-virus infections of the CNS. Nat Rev Immunol.

[13] Poli A, Kmiecik J, Domingues O, Hentges F, Blery M, Chekenya M, Boucraut J, Zimmer J (2013). NK cells in central nervous system disorders. J Immunol.

[14] Liu T, Chambers TJ (2001). Yellow fever virus encephalitis: properties of the brain-associated T-cell response during virus clearance in normal and gamma interferon-deficient mice and requirement for CD4+ lymphocytes. J Virol.

[15] Wang Y, Lobigs M, Lee E, Mullbacher A (2003). CD8+ T cells mediate recovery and immunopathology in West Nile virus encephalitis. J Virol.

[16] McCandless EE, Zhang B, Diamond MS, Klein RS (2008). CXCR4 antagonism increases T cell trafficking in the central nervous system and improves survival from West Nile virus encephalitis. Proc Natl Acad Sci U S A.

[17] Teo TH, Lum FM, Claser C, Lulla V, Lulla A, Merits A, Renia L, Ng LF (2013). A pathogenic role for CD4+ T cells during Chikungunya virus infection in mice. J Immunol.

[18] Johnson RT (1983). Pathogenesis of La Crosse virus in mice. Prog Clin Biol Res.

[19] Janssen R, Gonzalez-Scarano F, Nathanson N (1984). Mechanisms of bunyavirus virulence. Comparative pathogenesis of a virulent strain of La Crosse and an avirulent strain of Tahyna virus. Lab Invest.

[20] Pekosz A, Griot C, Stillmock K, Nathanson N, Gonzalez-Scarano F (1995). Protection from La Crosse virus encephalitis with recombinant glycoproteins: role of neutralizing anti-G1 antibodies. J Virol.

[21] Blakqori G, Delhaye S, Habjan M, Blair CD, Sanchez-Vargas I, Olson KE, Attarzadeh-Yazdi G, Fragkoudis R, Kohl A, Kalinke U (2007). La Crosse bunyavirus nonstructural protein NSs serves to suppress the type I interferon system of mammalian hosts. J Virol.

[22] Taylor KG, Woods TA, Winkler CW, Carmody AB, Peterson KE (2014). Age-dependent myeloid dendritic cell responses mediate resistance to la crosse virus-induced neurological disease. J Virol.

[23] Butchi NB, Woods T, Du M, Morgan TW, Peterson KE (2011). TLR7 and TLR9 trigger distinct neuroinflammatory responses in the CNS. Am J Pathol.

[24] Gangappa S, Deshpande SP, Rouse BT (1999). Bystander activation of CD4(+) T cells can represent an exclusive means of immunopathology in a virus infection. Eur J Immunol.

[25] Shrestha B, Diamond MS (2004). Role of CD8+ T cells in control of West Nile virus infection. J Virol.

[26] Bien CG, Bauer J (2005). T-cells in human encephalitis. Neuromolecular Med.

[27] Burdeinick-Kerr R, Wind J, Griffin DE (2007). Synergistic roles of antibody and interferon in noncytolytic clearance of Sindbis virus from different regions of the central nervous system. J Virol.

[28] Hooper DC, Morimoto K, Bette M, Weihe E, Koprowski H, Dietzschold B (1998). Collaboration of antibody and inflammation in clearance of rabies virus from the central nervous system. J Virol.

[29] Kitamura D, Roes J, Kuhn R, Rajewsky K (1991). A B cell-deficient mouse by targeted disruption of the membrane exon of the immunoglobulin mu chain gene. Nature.

[30] Mombaerts P, Iacomini J, Johnson RS, Herrup K, Tonegawa S, Papaioannou VE (1992). RAG-1-deficient mice have no mature B and T lymphocytes. Cell.

[31] Brandstadter JD, Yang Y (2011). Natural killer cell responses to viral infection. J Innate Immun.

[32] Dresser DW, Mitchison NA (1968). The mechanism of immunological paralysis. Adv Immunol.

[33] PrabhuDas M, Adkins B, Gans H, King C, Levy O, Ramilo O, Siegrist CA (2011). Challenges in infant immunity: implications for responses to infection and vaccines. Nat Immunol.

[34] Wekerle H (2002). Immune protection of the brain—efficient and delicate. J Infect Dis.

